# Warming Houses

**Published:** 1860-01

**Authors:** 


### WARMING HOUSES.

THE subject of keeping family dwellings healthfully and agreeably heated in winter-time is of great practical importance to every reader, and has long engaged the earnest and protracted study of the most inventive minds in the country. How to have a comfortable heat with the least waste of fuel, is a question which involves the health and lives of thousands, and millions of money every year.

There is no heat more soothing, more cheerful, more delightful, than that of a wood-fire in an old-fashioned fire-place, broad, deep, high and capacious. Most minds run back lovingly to the times when the then unappreciated wood-fire was the rule—the stove, the grate, the exception. But wood-fires in capacious fire-places are now a pecuniary impossibility to the masses in cities, and more and more so every day, even in the country. Various patterns of stoves have been devised as a means of saving wood, but unless wisely managed, and with constant attention, it is difficult to regulate the heat, and besides, there is a "closeness" which is disagreeable to all, and to some unendurable. But even a wood-fire in a stove is an expensive luxury, costing twenty-five dollars in New-York for a single good-sized room for the winter, while that amount expended in coal would answer all the domestic purposes of many families. A common grate for anthracite coal sends full two thirds of its heat up the chimney to warm all out-doors, thus thirty dollars' worth of coal yields but ten dollars' worth of warmth; this is the calculation of scientific men. To remedy this waste, and to save the trouble of kindling and keeping up half a dozen different fires in the same household, stoves have been devised, intended to be large enough for a whole house; these are placed in the cellar where one kindling does for all, and for a week or month together, with a happy deliverance from the dust and ashes and trouble attending a multitude of separate fires. These immense stoves for the cellar are called furnaces, the heat from which is conducted to any desired part of the building, through tin tubes which are built into the wall, while the house is in course of erection. These furnaces with their pipes and registers, are in important respects highly objectionable.

In the first place, either owing to the parsimony of landlords, or to the ignorance of contractors and builders and architects, they are constantly burning down houses, public and private, as well in Philadelphia as in New-York.

A second objection is, that a great deal of heat is conveyed to the upper parts of the building, and to rooms where heat is not wanted, when it is indispensably needed on the first and second stories.

Thirdly, no furnace yet known, keeps a good-sized house comfortably warm in the severest weather, unless by such a ruinous consumption of fuel, or danger of conflagration, that persons prefer calling in the aid of the grate, when the thermometer hugs zero, or comes within a dozen degrees of it, so that every furnace yet devised is an acknowledged failure.

A fourth objection to furnace heat, is perfectly fatal to its wise adoption. The air which comes in contact with the furnace, is burnt, it is in part decomposed, and is no longer fit for purposes of healthful respiration. Whenever air comes in contact with a nearly or quite a red-hot metallic surface, it is no longer fit to enter the lungs of any thing that breathes, and is instantly detected by the feeling which is expressed by the term "closeness." The air has in it such a small amount of living sustenance, that an ordinary quantity taken through the nostrils is not enough, and the person instinctively opens the mouth, literally gasps for more, as a fish for water when thrown out of its native element.

Almost every furnace inventor will tire you with reasons why his furnace does not burn the air, but any man's nose gives the flat contradiction. Besides, all these cellar furnaces are perfect maelstroms of fuel; they lick it up as the flame licks up water.

Another plan has been devised, and is in successful operation in the Breevort House on Fifth avenue, and in prisons and insane asylums. Hot water is conveyed in pipes from the cellar to every part of the house; this certainly gives an equable and balmy warmth, and is second only to a wood-fire in the old time fire-place. But it is too expensive for general adoption; besides, the pipes may commence leaking at any one of their hundreds of joinings, and the building drenched with hot water at any hour of the day or night.

Under all the circumstances of the case we think the fire which warms our office at this present writing, when the whole air is filled with driving snow, and Farenheit is below the freezing-point, is, next to the old-fashioned wood-fire of forty years ago, the very best ever devised, as we think any intelligent observer will see in a moment, if he chooses to call. It is simply common anthracite coal burning flat on the hearth, in a fire-place nearly a yard across, with oval back and flaring jams, which necessarily throw the heat out into the room. As evidence, the thermometer five feet from the floor and twelve feet from the fire, on the wall opposite, is at this moment above seventy degrees, and has been in that neighborhood since the early morning; the fire having been made at daylight and never touched since, except to lay on a few pieces of coal about two o'clock, and no more will be needed for the remainder of the day and evening; in other words, without more coal, our office will be comfortable until near midnight, so we are informed, for we trot off to bed at nine, and can not speak from personal knowledge.

But what is the quality of this heat? According to our best judgment and remembrance, it is as balmy as that of the universal favorite, a wood-fire. But how can that be, when coal has no oxygen, and without oxygen it can not burn at all, and must get it from the air of the apartment where the fire is, producing the "closeness" which belongs to all furnace heat? It happen in this wise, all the premises are true, except one. The oxygen is not supplied from the air of the room, but from the cellar; hence the air of the apartment is simply pure air warmed.

Another advantage is, this fire is kindled without the troublesome and unsightly "blower," and the ashes are taken up but once a year, for they fall through crevices in the hearth into a close brick receptacle in the cellar without any possibility of contact with any combustible material; hence the flying dust of ashes inseparable from the cleaning of a grate is avoided. The deposit on the mantle from a whole day's burning is scarcely observable, for a poker is never needed. After all, the prime consideration with many, is the cost of the fuel, in comparison with other modes of heating apartments. Without troubling the reader with statistical tables, a few figures will be given in connection with this low-down grate.

We have weighed and used to-day, and on several similar days, very near fifty pounds of coal, the thermometer without having been steadily below the freezing-point, while it has stood about seventy in our office in the position before described. Hence fifty pounds of coal will keep a room two hundred and forty square feet rather too warm for comfort and health when it is freezing out of doors, and at a cost of five dollars a ton, (placed in bins in the cellar,) two thousand pounds being a New-York retail ton, or twenty-five bushels of eighty pounds each, one pound of coal costs a quarter of a cent, or twelve and a half cents for fifty pounds, or eighty-seven and a half cents a week, three dollars and three quarters a month, or twenty-two dollars and a half for the season of six months, supposing that each day was freezing cold without, but there are not thirty such days during any winter, and from observation we think that thirty pounds a day would be an ample average, or seven and a half cents a day, fifty cents a week, or thirteen dollars for the winter.

For a family apartment, in ordinary cases, the thermometer should not be higher than sixty-five, twelve feet from the fire; more than that debilitates, and less is too cool for children and persons at rest.

We must say in addition, in praise of the "low-down" grate, that when the thermometer is at twenty, its broad bed of flaming coals, two and a half feet across, with the soft and soothing atmosphere of the apartment, is cheery to the sight and to the sensations most delicious, and as such a grate costs from thirty to fifty dollars, when there are no extra attachments, we heartily commend it to public attention.

The judgment of the observant is rapidly settling down in the conviction that furnace-heated houses are rapidly undermining the constitutions of whole families, and thus render them the easy prey to every acute disease. Such being the case, it is literally a matter of vital importance to discover a remedy or a substitute, to discover some method of heating a family apartment in such a manner as will combine regularity of temperature, sufficiency of heat and adequate ventilation, with an economy of fuel adapted to the means of the masses. It is believed that any one who will call at the Editor's office will be convinced that the low-down grate is one of the greatest improvements yet introduced for the healthful warming of family apartments. It is, without exaggeration, really difficult to point out a single defect, or offer a single well-grounded objection to this invention, so new to New-York, yet known to a neighboring city for seven years.

